# A deep learning pipeline for age prediction from vocalisations of the domestic feline

**DOI:** 10.1038/s41598-025-17986-z

**Published:** 2025-10-03

**Authors:** Astrid van Toor, Nadeem Qazi, Stefania Paladini

**Affiliations:** 1 Present Address: blueOASIS, Ericeira, Portugal; 2 Computer Science and Digital Technologies, University of East London, University Way, London, E16 2RD England; 3 Business School, Queen Margaret University, Queen Margaret University, Drive, Musselburgh, EH21 6UU Scotland

**Keywords:** Age estimation, Digital bioacoustics, Animal vocalisation, Transfer learning, Deep learning, Computational biology and bioinformatics, Ecology, Ecology, Zoology

## Abstract

Accurate age estimation is essential for advancing interspecies communication but remains a challenge across non-human species. This study presents the first dataset of domestic feline vocalisations specifically designed for age prediction and introduces a novel deep learning pipeline for this purpose. By applying transfer learning with models like VGGish, YAMNet, and Perch, we demonstrate the potential for automated age classification, with VGGish achieving the best results. Our findings hold significant potential for applications in veterinary care and wildlife conservation, building on existing research and pushing forward the boundaries of automated age classification within digital bioacoustics. Future work could explore improving model generalisability and robustness, potentially expanding its application across species.

## Introduction

Humans and cats have had a mutual tolerance for millenia^1^ and with an estimated 600 million cats living with humans worldwide^2^ they are one of the most common companion animals. Despite this, we are still trying to understand the communication habits of our beloved furry friends. Studies from the last decade have made progress in deciphering cats’ ability to recognise individual humans^3^ and their own names^4^, as well as their use of multimodal signals in interactions with humans^5,6^. Yet, the relationship between vocalisations and age in cats remains underexplored, despite its potential significance for veterinary care, animal welfare, and conservation.

This study introduces the first application of deep learning techniques to non-human vocalisations for age estimation, focusing specifically on domestic felines - hereafter referred to as feline or cat. Using transfer learning, we developed a novel, non-invasive method for predicting a cat’s age and collected the first publicly available dataset tailored for this purpose. The key motivations and contributions of this work are summarised below:

- Age plays an important factor in identifying different types of vocalisations^7^, crucial in furthering our understanding and protection of non-human species.
- Despite advances in human vocal age estimation^8,9^, automated age prediction from non-human vocalisations remains underexplored.
- We collected and processed the first publicly available dataset of cat vocalisations tailored for age prediction.
- We built a deep learning pipeline for auditory age estimation that is adaptable to other species.
- We applied and evaluated prominent transfer learning models for audio analysis (VGGish, YAMNet, Perch) for feature extraction in this novel context.
- We present a comparative analysis of state-of-the-art transfer learning models in age prediction from vocalisations.

The remainder of this paper is organised as follows: Background and research context reviews existing literature and provides research context of where our study fills a gap. In Methodology, we describe the methodology, including data collection, audio input requirements, feature extraction, and the optimisation and validation processes. Results and discussion presents the results of this study, followed by a discussion of limitations and future research directions in Conclusion and limitations.

## Background and research context

### Age-related changes in vocalisations

Research has shown that vocalisations, particularly the fundamental frequency (F0), change with age in cats^10–12^, suggesting a potential indicator for automated age classification. Similar age-related vocal changes by F0 reduction have been observed in other species such as elephants^13^, cheetahs^14^, howls^15^, fissiped carnivores^16^, wolves^17^, deer^18^, primates such as langurs and baboons^19^, as well as humans^20,21^. Other species such as pigs^7^, whales^22^, bats^23^, and the leopard seal^24^ also demonstrate age-related shifts in their vocal frequencies or other acoustic features.

These findings suggest that vocal patterns related to age are a widespread phenomenon across species. However, the application of automated techniques - particularly using deep learning - to classify age from vocalisations remains largely underexplored in non-human animals, despite successful applications in humans^8,9^. Deep learning, which has been effective in recognising specific cat vocalisations^25,26^, offers a promising approach for age classification. While human age estimation benefits from large datasets, limited non-human vocal data necessitates the use of transfer learning to capture age-related acoustic differences. Looking at gender over age,^14^ found significant differences in fundamental frequency between male and female cheetahs and suggest this may be applicable to domestic cats due to their inherent similarities. While this is an interesting direction, gender analysis is largely out-of-scope in this study due to data limitations.

### The role of transfer learning

Transfer learning allows models that are pre-trained on large, diverse datasets to be adapted to specific tasks where data is limited - such as age estimation in non-human species. This method is particularly powerful in audio analysis as it enables the model to extract meaningful features from for example cat vocalisations without the need for massive datasets. In our study, we leveraged well-known pre-trained models for audio classification tasks—specifically VGGish^27,28^, YAMNet^29^, and Perch^30^—to address the data limitations that are common in animal vocalisation research. These models were selected due to their established success in audio analysis tasks^25,31–37^, but their application in age estimation from vocalisations had not yet been explored.

To address this gap, our study focused on evaluating the effectiveness of transfer learning models for classifying age based on vocalisations in a case study of the domestic feline. Drawing on the success of machine learning and statistical methods in estimating age for species like elephants^13^ and cheetahs^14^, we hypothesised that fundamental differences in the frequencies and characteristics of a cat’s meow could be used to classify felines into different age groups. Table 1 summarises works involving prediction models for age classification from vocalizations across species, reflecting the limited exploration of automated techniques in non-human animals.

By using VGGish, YAMNet, and Perch to extract acoustic features from our dataset, we developed a downstream deep learning model (multi-layer perceptron (MLP)) to predict a cat’s age from its vocalisations. This study employed the use of advanced artificial intelligence tools to better understand the communicative and biological signals in cat vocalisations, setting a precedent for future research at the intersection of machine learning and animal behaviour. To support this, we collected a novel and diverse dataset of cat vocalisations from public contributors, ensuring sufficient data quality and accurate annotations.

A key benefit of using VGGish, YAMNet, and Perch in our study lies in the comparative review this approach offers. While each model has demonstrated competitive results in various domains, their relative strengths vary by task. VGGish has excelled in tasks such as song segment classification^36^ and cardiovascular disease detection^33^, while YAMNet outperformed VGGish in applications of passive acoustic monitoring (PAM) in wildlife sound detection^35^ and marine bioacoustics^37^. However, recent studies have shown that Perch significantly outperforms both models in a range of bioacoustics classification tasks for species detection^31,37^. This comparison underscores the robustness of Perch, making it an essential addition to our study.

### Study significance and applications

The development of a non-invasive method for identifying a cat’s age through vocalisations could have far-reaching implications. In veterinary care, it could aid in tailoring treatment plans based on a cat’s age. Rescue centres could benefit from creating more suitable adoption profiles. Additionally, this research could contribute to a better understanding of age demographics in domestic and feral cat populations, aiding research on genetics, behaviour, and conservation especially for feral or community cat populations.

Beyond the domestic feline, the successful implementation of such a pipeline could be extended to other species, opening up new avenues for research and innovation in animal care, behaviour, and conservation. As interest in the phonetics, social structures, and “language” of non-human species continues to grow, tools for vocal age estimation could play a prominent role in advancing our understanding and protection of animals.

Notably, gender and age data are commonly used to set harvest guidelines, observe the demographic composition, health, and viability of populations, and offer insights into behavioural ecology - crucial for wildlife monitoring^15,38,39^. While gender is often more easily observable from video material, to determine age is at its core, for many species, a highly manual and invasive effort or it relies on findings such as wings, jaws, or teeth^38,40,41^. Being able to automatically infer age from sound recordings through techniques like PAM would be a pivotal advancement in conservation. Demonstrated previously by^24^; this study on marine animals showed that using PAM to monitor age cohorts via F0 reveals age related differences in calling rates, reflecting habitat quality and population dynamics. Such methods offer valuable insights into ecological changes and environmental impacts on the species being monitored.

Furthermore, age estimation plays an important role in interspecies communication;^7^ discovered a variation of sound attributes over different age groups in pigs, meaning acoustic attributes vary so significantly that there is not one call for distress that is generalisable over all ages. When considering the case from a human perspective, this makes sense; a human child in distress makes a vastly different sound than a human adult - it is just the understanding that comes naturally to us.

This is where our research plays a valuable role, as we aim to lay the groundwork for an age estimation pipeline capable of adapting to the unique acoustic characteristics of various species. In cats specifically, there is a clear need for research to advance knowledge of feline vocalisations and gain insights into the cat’s emotional states and needs^5,42,43^. Beyond the study of feline vocalisations, the methods developed here could be applied to better understand the emotional well-being of other animals, including those in industrial farming settings. Improved communication models may enable more accurate monitoring of animal welfare, potentially informing policies that could reduce harmful practices. Such developments would not only have ethical implications but also benefit public health and environmental sustainability^44^.

**Table 1:** Summary of studies on age prediction by vocalisation across species.

Animal Species	Study	Vocalisation Type	Features Analysed	Methods/Models Used	Sample Size	Key Findings	Accuracy / Performance
Pigs	^7^	Various (Distress-related)	Signal energy, duration, pitch, max/min amplitudes, intensity, formant frequencies	ML Decision Tree	40 pigs (20 males, 20 females)	Vocalisation features correlated with sex, age, and distress in pigs.	Distress call classification with 81.92% accuracy
Humans	^8^	Speech	MFCC, Chroma, Rasta-PLP, and others (450 features total)	Multi-Layer Perceptron (MLP)	71k samples split over 8 age groups (Mozilla Common Voice dataset)	Accurate classification across 8 age groups; highest observed accuracy in the literature for age estimation with deep learning models - likely contributed by availability of large-scale dataset.	94.34% accuracy
Humans	^9^	Speech	MFCC, x-vector (TDNN feature extraction), d-vector (LSTM feature extraction)	DNN-based architecture	180k samples split over 8 age groups (Common Voice, VoxCeleb, TIMIT dataset)	Advanced deep learning models achieved state-of-the-art age estimation accuracy; MAE of 5.12–5.29 years for males and females respectively.	MAE: 5.12 (males), 5.29 (females)
Cheetahs	^14^	Chirps	Fundamental frequency (f0max), acoustic parameters	Polynomial Regression Models	56 litters split to 14 age classes	f0max decreases steadily with age; polynomial model effective for age estimation, particularly for cheetahs under 4 years old.	Model predicts age of individuals with precision of ±4.3 months and is particularly effective at distinguishing young cheetahs to mature cheetahs.
African Elephants	^13^	Rumbles	Fundamental frequency, formant frequencies, harmonics, MFCC, GFCC	ANOVA, PCA, Discriminant Analysis (DA), and automated classification with SVM, NN, and LDA	526 rumbles from 101.4 hrs of recordings split into four age groups	Age groups discriminated effectively; high classification accuracy for distinguishing adult vs. infant/calf categories.	95% (binary); 70% (4 age groups)

**Fig. 1:**
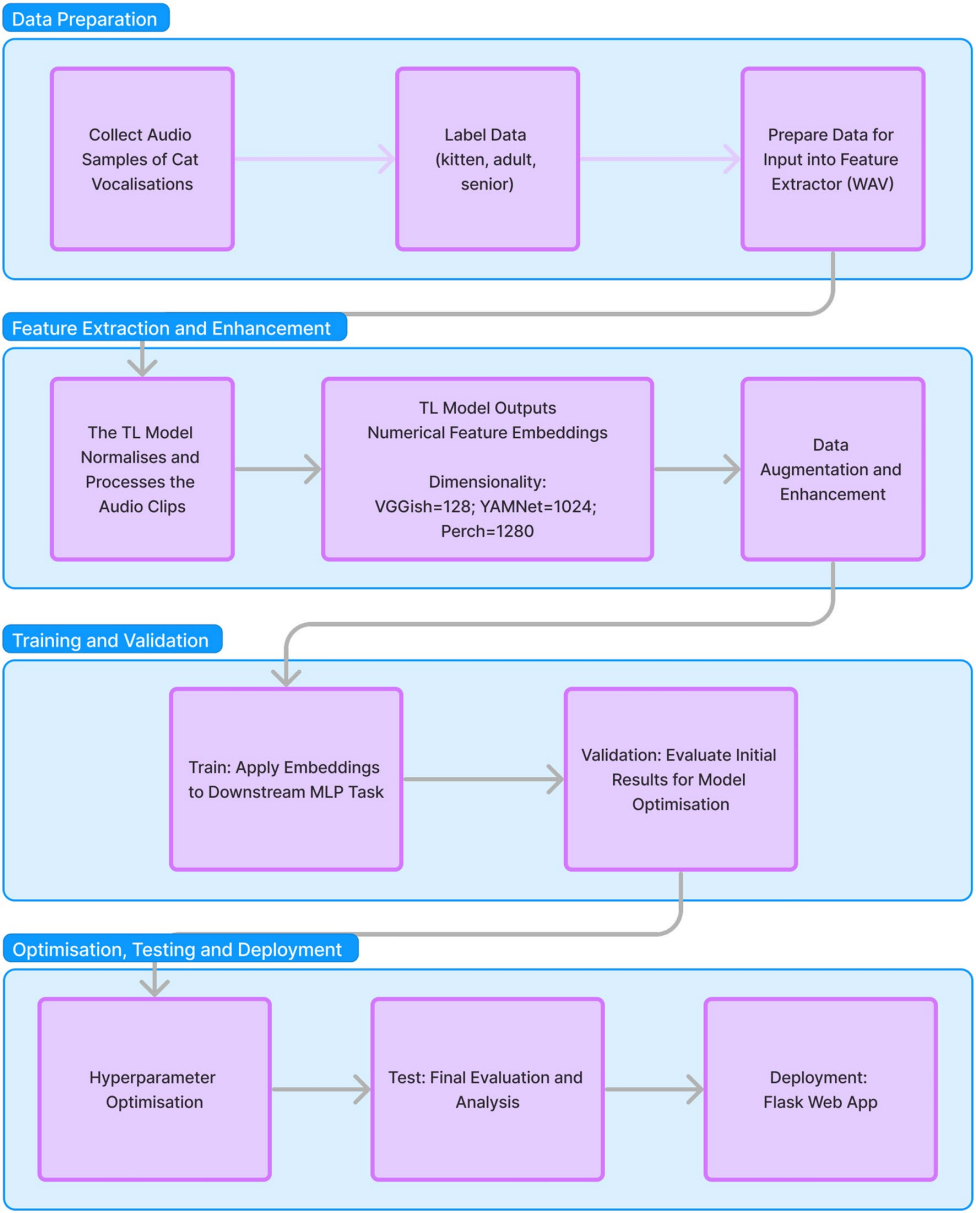
Methodology pipeline.

## Methodology

### Research design and rationale

This study employed a deep learning approach to age estimation in domestic cats based on vocalisations. The methodology integrated both data collection and model development combined with transfer learning models for feature extraction and downstream classification tasks. Given the lack of a publicly available dataset suitable for this task, we conducted data collection and explored existing datasets for potential inclusion. Following data collection, we applied transfer learning models (VGGish, YAMNet, Perch) for feature extraction, and processed the resulting embeddings through an MLP neural network for classification. The following sections describe in detail the data collection approaches (see Data Collection, Data Context Restrictions, and Data Sharing), feature extraction through transfer learning (Transfer Learning Models for Feature Extraction, and Audio Input Requirements for VGGish, YAMNet, and Perch), the preparation of feature embeddings for downstream learning (Preparing Feature Embeddings for Downstream Learning), hyperparameter tuning and bias mitigation (Hyperparameter Tuning and Bias Mitigation), data enhancement techniques (Data Enhancement), and an ethics declaration (Ethics Declaration). Figure 1 provides an overview of the full methodology pipeline.

### Data collection

Due to the lack of a suitable available dataset, original data collection was conducted. The initial consideration for this research was to, partly, use the CatMeow (Ludovico et al., 2020) dataset. Unfortunately, this dataset was recorded using low-budget Bluetooth devices, which, while making the data collection accessible and scalable, compromised the audio quality. Problems such as white noise and poor sound quality overall, including interruptions in the waveforms, led to the decision to exclude this dataset from our study. We reached out to the owners of two other datasets;^45^ had not recorded ages and^11^ could not yet publicise due to ongoing research. The dataset presented in this work is the first publicly available dataset of its kind.

For the data collection we reached out to online communities through platforms such as Reddit, Lemmy, TikTok, LinkedIn, Instagram, and local communities through personal networks, cafes, and veterinarians, supplemented by information leaflets and promotional videos. A significant amount of contributions were sourced through Lemmy and freesound.org, each harbouring a supportive and engaged community.

From freesound.org, only lossless recordings in either WAV, FLAC, or M4A were selected, ensuring audio quality was of a high standard. The required information for the samples, such as the cat’s ages, was acquired by contacting the owners directly. The remaining participants were instructed to use the ’Dolby On’ recording app (no affiliation), specifically set to Lossless audio in WAV format at 48kHz, to ensure uniformity and optimal audio quality when recording from mobile devices. Individual submissions in other formats or of poor quality were discarded. The majority of samples collected were at 48kHz (63%), followed by 44.1kHz (24%), and some at 96kHz (7%) and 16kHz (6%), all in 16-bit audio and transformed to Waveform Audio File Format (WAV) to fit the expected input format of the transfer learning models. While the unique recording settings for each contribution may enhance real-world generalisation, in the context of a smaller dataset this could become a disadvantage. The model has fewer environmentally consistent examples to learn from, which may hinder performance. However, we prioritised generalisation over potentially over-optimistic results.

**Table 2:** Dataset summary and demographic distribution.

Metric	Value
Total audio clips	793
Unique cats	111
Age range (years)	0.00 – 18.00
**Sex Distribution**	
Female (F)	231 (29.1%)
Male (M)	286 (36.1%)
Unknown (X)	276 (34.8%)
**Clips per Age Group**	
Kittens (0–0.5 years)	135 (17.0%)
Adults (0.5–10 years)	405 (51.1%)
Seniors (10+ years)	253 (31.9%)
**Average Age per Group (years)**	
Kittens	0.0
Adults	4.47
Seniors	13.31
**Vocalisation Duration (seconds)**	
Shortest	0.08
Longest	4.42
Mean	0.72
**Sex by Age Group**	
Kittens – F/M/X	0 (0.0%) / 0 (0.0%) / 135 (100.0%)
Adults – F/M/X	134 (33.0%) / 178 (44.0%) / 93 (23.0%)
Seniors – F/M/X	97 (38.3%) / 108 (42.7%) / 48 (19.0%)

F=Female gender, M=Male gender, X=Unkown gender.

### Data context restrictions

Inclusion was restricted to those cases where owners had certainty over their pet’s age. Breed information was generally not collected due to many owners’ uncertainty about their pets’ specific breeds. In some samples contexts were noted including vets visits, food, attention, and door. Some users provided valuable time-series data from the same cats over different years - ideal for our task. For the purpose of direct comparison and due to data limitations, types of vocalisations included in this research include meows (a sound that often includes multiple vowels), mews (higher pitched meows), and squeaks (short raspy, nasal, high-pitched mew-like call) as defined by^46^ and later expanded by^10^. Vocalisations such as purrs and yowls were therefore discarded. Noisy samples or those with overlapping sounds were also discarded. Litters of kittens could not be uniquely identified so were grouped together.

A total of 793 meows were included, each manually extracted using Audacity to ensure the highest quality. In some cases, a noise section of otherwise good quality meows were cropped out - ensuring each cat had at least one full meow. This modification does mean that vocal duration cannot be taken into account in this study. A statistical summary of the dataset is presented in Table 2.

In our dataset the youngest cat is five weeks old while the oldest cat is eighteen years old. Considering the relatively small dataset, it was decided to focus on three age categories for classification: kittens, adults, and seniors. Since there is no clear consensus on when an adult cat transitions into a senior, the age ranges were defined as follows: kittens (0 - 0.5 years), adults (0.5 - 10 years), and seniors (10+ years). These ranges are based on typical developmental stages in cats where kittens represent early life stages, adults cover the majority of a cat’s mature lifespan, and seniors reflect the later years. This categorisation results in 135 kittens, 405 adults, and 253 senior cats, leading to an imbalanced dataset. Finally, each cat is assigned a unique identifier that allows us to group cats together during training and testing, avoiding data leakage.

### Data sharing

There is a lack of open source data in bioacoustics^47^; just 21% of publications in the field publish their recordings for further research^48^. The dataset compiled for this study will therefore be made available alongside the publication, ensuring that future researchers can access high-quality audio samples for further analysis.

### Transfer learning models for feature extraction

As discussed, VGGish, YAMNet, and Perch have been proven to be effective in a number of wide-ranging applications for sound detection^25,31–37^. Their open accessibility and comprehensive documentation facilitates straightforward use, enabling replicability and potential for further development. All models automatically convert raw input audio in WAV format to spectrograms for feature extraction into high-dimensional vector embeddings; numerical representations of the data. A summary of these models along with precise input requirements and restrictions are discussed as follows.

VGGish is modified from the VGG Convolutional Neural Network (CNN) - characterised by its simplicity, depth, the use of small convolutional filters, and known for top performance in image classification tasks^49^. YAMNet is another CNN-based deep neural network, utilising the MobileNet V1 architecture - known for its efficiency and effectiveness, particularly in environments with limited computational resources^50^. Perch contains an EfficientNet B1 backbone - a CNN designed for high efficiency and scalability across different computing environments^51^. While VGGish was trained on YouTube-8M (closely related to AudioSet) and YAMNet directly on the AudioSet dataset^52^, both of which focus on general sound events, Perch was specifically trained on birdsong embeddings derived from XenoCanto wildlife data. An overview of the three architectures is presented in Table 3.

All models can either be run locally or through hosted versions on TensorFlow Hub (TFHub). VGGish performed better locally using TensorFlow’s Model Garden^53^, possibly due to system configurations or outdated versions on TFHub. The core dependencies for this local setup can be found in Table 4. For YAMNet, TFHub inference^54^ was found more efficient than the Model Garden build with minimal performance difference, so this option was chosen. Perch, lacking public pre-trained network weights and requiring substantial computational resources, was not built locally therefore also used TFHub inference^55^.

**Table 3:** Comparison of neural network architectures for audio feature extraction.

Network	Architecture	Training Data	Input Sample Rate (kHz)	Embedding Window (s)	Embedding Dimension (Output)	CPU (ms/s) $^{1}$
VGGish	VGG	YouTube 8M	16	0.96	128	2.8
YAMNet	MobileNet v1	AudioSet	16	0.96	1024	7.7
Perch	EfficientNet B1	XenoCanto	32	5	1280	24.3

$^{1}$Source:^31^ benchmarked CPU performance on a 4.3 GHz AMD CPU with 12 cores.

**Table 4:** System configuration for VGGish environment.

Component	Details
System	Darwin
Release	21.6.0
Machine	arm64
Python Version	3.10.13
pandas	2.1.4
numpy	1.23.2
tensorflow	2.13.0
Total RAM (GB)	16.0
Number of GPUs	0

This configuration was used for benchmarking VGGish.

### Audio input requirements for VGGish, YAMNet, and Perch

This section describes the input requirements for each architecture based on model documentation and codebases. We found that looping the audio data to fit the required embedding window size (Table 3) was preferable to padding it with silence. All models normalise the audio to a range of -1.0 to +1.0 and apply a Short-Time Fourier Transform (STFT) to generate the spectrogram. The STFT is a technique that transforms the audio signal from the time domain to the frequency domain, enabling the analysis of frequency properties of sound signals. By segmenting the signal into small overlapping time windows, the STFT allows for a detailed analysis of frequency variations over time, which is represented visually as a spectrogram.

#### VGGish

VGGish accepts raw WAV files for feature inference and generates feature embeddings in 0.96-second intervals. For this study, we modified the sample window hop size to 0.48 second (from 0.96 seconds) to ensure a 50% overlap in audio processing, aligning with YAMNet’s pipeline. The audio is looped using Pydub to fit these increments. VGGish resamples the audio to 16kHz mono, normalises it, and computes a spectrogram using STFT with a window size of 25ms and 10ms hop size. This results in overlapping frames of 25ms each. The spectrogram is then mapped to 64 mel bins, which represent frequency bands on a scale that more closely matches how humans perceive sound. This scale emphasizes lower frequencies and compresses higher ones, making it well-suited for analysing vocalisations. In this case, the bins cover a frequency range from 125Hz-7500Hz, which is sufficient for cats but may need adjustment for other species. Finally, a log function was applied to produce a log mel-frequency spectrogram, and the resulting features passed to VGGish’s neural network model to generate the numerical embeddings.

#### YAMNet

Developed by the same authors as VGGish, YAMNet follows an identical input process to VGGish, with the same STFT configuration and audio sampling.

#### Perch

Perch processes audio in significantly longer 5-second segments and uses a 32 kHz mono sampling rate, double the rate of VGGish and YAMNet. A small proportion of samples (6%) were upsampled to match this rate, though this is expected to have minimal impact since they represent just two cats. While it is known that Perch uses STFT to create spectrograms, further configuration details are not disclosed in the TFHub documentation so we can not make any further statements on the internal processing.

### Preparing feature embeddings for downstream learning

One of the benefits of using pre-trained models as feature extractors is that it simplified data processing; the transfer learning models internally normalise and process audio clips. After feature extraction we passed the embeddings to an MLP neural network, a type of artificial neural network mimicking the inner workings of the human brain and one of the most commonly used neural networks^56^.

For this downstream task, data standardisation and scaling is important to ensure that all features contribute equally to model training. We applied Scikit-learn’s StandardScaler to ensure features have a mean of zero and a standard deviation of one, which helps improve the training stability and performance of models like MLPs by ensuring that each feature contributes equally to the learning process. Other scalers like RobustScaler and MixMaxScaler were explored, but StandardScaler showed best initial results. Further tuning may be needed.

### Hyperparameter tuning and bias mitigation

The downstream models were developed with TensorFlow’s Keras, a well known end-to-end open source machine learning framework. To determine the best configuration, we used the Optuna hyperparameter tuner for its ease of implementation. For each model, VGGish, Perch, and YAMNet, 300 trials were run to find the optimal parameters. The depth of our exploration is detailed in table 5.

**Table 5:** Hyperparameters and their search space for tuning with Optuna.

Hyperparameter	Search Space
Learning Rate	$\text {log-uniform}(1e{-4}, 1e{-2})$
Optimizer	{Adam, Adamax, RMSprop}
Batch Size	{16, 32, 64}
Activation Function	{relu, sigmoid}
Number of Layers	{1, 2, 3, 4}
Units per Layer	{32, 64, 128, 224, 256, 480, 512} for each layer
Dropout Rate per Layer	$\text {uniform}(0.1, 0.5)$ for each layer

#### Stratified sampling and grouping

Stratified sampling is a technique to ensure that samples in both the train and test set are selected in the same proportion^57^, crucial for handling our imbalanced dataset. To prevent data leakage between the train and test set, we grouped samples by cat_id using Scikit-learn’s StratifiedGroupKFold, which allowed for stratification while ensuring no overlap between splits. This technique ensured that all samples from each cat_id appeared in the test set exactly once across k-fold validation rounds. With limited data, 4 folds was the limit without causing severe class imbalances.

One adaption to the data distribution after splitting is that adult cat 000A and kitten 046A were always included in the training set, swapped out for a random cat_id of the same class when necessary. This decision was made because these cats in particular contributed a high number of samples and over varying contexts, containing valuable information crucial for learning performance. Lastly, to avoid patterns in the data that could be learned by the model, we shuffled each class’s samples once before splitting and once right before model training.

#### Avoiding overfitting and bias

**Fig. 2:**
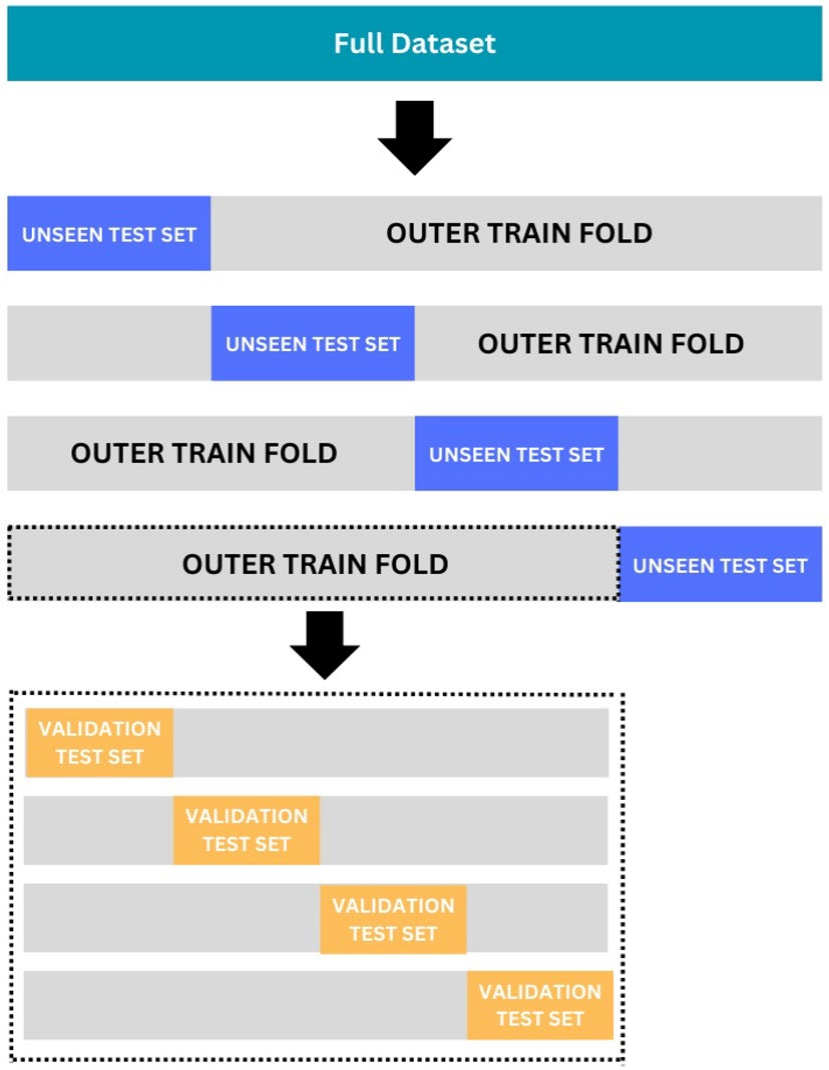
Schematic of the nested cross-validation method used in our study. The dataset is divided into four outer folds, each containing a distinct test set and a corresponding training set. Each training set is further divided into four inner folds used for validation of the hyperparameters during Optuna’s tuning process.

Dropout layers^58^ (Keras function) and an early stop function^58^ (Keras function) were used to minimise overfitting. To further minimise bias and to obtain a robust statistical performance estimate, we applied nested cross-validation^59^. This technique, visualised in Fig. 2, allowed us to tune hyperparameters on a validation set in the inner loop while using an unseen test set in the outer loop for final evaluation. This prevents tuning bias and allows a fair comparison between architectures.

Each Optuna trial included 4 outer and 4 inner loops (16 runs total), where Optuna optimised the next set of parameters based on the average F1-score in our inner loop. Each set of hyperparameters is therefore optimised without direct knowledge of the outer test data, which it would eventually be evaluated against.

**Table 6:** System configuration for downstream learning after obtaining the feature embeddings from the respective transfer learning models: VGGish, Perch, and YAMNet.

Component	Details
System	Darwin
Release	21.6.0
Machine	arm64
Python Version	3.10.12
pandas	1.5.3
numpy	1.25.2
tensorflow	2.15.0
Total RAM (GB)	16
Number of GPUs	0

#### Challenges and adaptations

A limitation of this method is that subdividing an already small dataset in the inner loop further reduces data available for training, leading to poorer performance on the inner validation set. As a result, hyperparameter tuning in the inner loop does not always generalise well to the outer (test) set. While “better” hyperparameters could be obtained by tuning on the outer set, this would introduce bias as the model would adapt to the test data and would violate the assumption of unseen data^59^. Additionally, due to computational constraints we had to slightly adapt the cross-validation method as described; we averaged the F1-score across inner folds rather than tuning for each fold individually. While this may introduce a small amount of bias, we believe that the combination of nested cross-validation, multi-seed validation (discussed below), and the careful data-splitting and grouping by cat_id are the best practices for unbiased model evaluation and optimising. After determining the best parameters, we manually fine-tuned them to further optimise performance.

#### Performance metrics and testing for robustness

As we are dealing with imbalanced data, the formula for macro-averaging as per Scikit-learn’s model evaluation was applied to give equal weight to each class in performance calculations^60^, with a focus on the F1-scores for comparison.

$$\text {Macro-}M = \frac{1}{|L|} \sum _{l \in L} M(y_l, \hat{y}_l)$$

Here, $\text {Macro-M}$ represents the macro-averaged metric. |*L*| denotes the number of classes. The summation $\sum _{l \in L}$ indicates a sum over each class. $M(y_l, \hat{y}_l)$ is the F1-score for a single class *l*, calculated from its true labels ($y_l$) and predicted labels ($\hat{y}_l$).

The F1-score is a statistical measure that balances the precision (ratio of correctly predicted positives to total predicted positives) and recall (ratio of correctly predicted positives to all actual positives). This single score helps us assess our model’s performance by identifying the accuracy of true positives. Finally, to determine robustness and reliability of the results to real-world application, we applied Levene’s test for statistical significance of variance over the aggregated metrics, as outlined by^61^.

Our final performance estimate applies standard 4 fold cross-validation with train/test split only - the validation set is left out to preserve more data for training, considering prior intensive validation during optimisation on the inner loop. The results are run over five different seeds to enhance reliability while ensuring reproducibility. The five seeds are randomly generated by another (reproducible) random seed - namely 42. The grouping by cat_id further contributes to a truer performance estimate as the test set will always contain samples recorded in settings different to the training samples; these novel conditions act as unseen real-world samples. The final performance estimates are averaged from cross-validation over seeds and folds to provide a robust estimate of performance on real-world data. The system configuration used for the downstream tasks in this project are detailed in Table 6. The hyperparameters used to train the MLPs with the extracted embeddings from each TL architecture, are detailed in Tables 7, 8, and 9.

**Table 7:** VGGish embeddings hyperparameters for downstream MLP task.

Parameter	VGGish Categorical	VGGish Binary
Learning Rate	0.00311	0.00311
Optimizer	Adamax	Adamax
Batch Size	128	16
Activation	relu	relu
Number of Layers	1	1
Units in Layer 0	128	128
Dropout in Layer 0	0.44571	0.44571
Output Layer	softmax	sigmoid
Output Size	3	1
Class Weights	Y	N

**Table 8:** YAMNet embeddings hyperparameters for downstream MLP task.

Parameter	YAMNet Categorical	YAMNet Binary
Learning Rate	0.00927	0.00927
Optimizer	Adamax	Adamax
Batch Size	32	32
Activation	relu	relu
Number of Layers	1	1
Units in Layer 0	480	480
Dropout in Layer 0	0.40037	0.40037
Output Layer	softmax	sigmoid
Output Size	3	1
Class Weights	Y	N

**Table 9:** Perch embeddings hyperparameters for downstream MLP task.

Parameter	Perch Categorical	Perch Binary
Learning Rate	0.00740	0.00740
Optimizer	Adam	Adam
Batch Size	32	32
Activation	relu	relu
Number of Layers	4	4
Units in Layer 0	512	512
Dropout in Layer 0	0.15689	0.15689
Units in Layer 1	64	64
Dropout in Layer 1	0.28869	0.28869
Units in Layer 2	128	128
Dropout in Layer 2	0.27322	0.27322
Units in Layer 3	32	32
Dropout in Layer 3	0.40641	0.40641
Output Layer	softmax	sigmoid
Output Size	3	1
Class Weights	Y	Y

### Data enhancement

In this study, various data augmentation techniques including pitch shift, time stretch, and gain manipulation (implemented using audiomentations) were applied across multiple configurations to balance class distributions and improve model performance. Detailed audiomentations results are omitted for brevity but are available upon request. Popular data augmentation techniques SMOTE and MixUp were also tested but failed to yield significant performance; these results are available in the supplementary code repository.

Despite the breadth of augmentation approaches, none demonstrated substantial improvement in classification performance. This suggests that these methods, while useful for specific contexts, might not be sufficient for enhancing performance in this case. Although our experiments did not show significant benefits from traditional augmentation methods, these observations highlight the importance of model experimentation and optimisation in digital bioacoustics and future work could seek to explore more advanced augmentation techniques such as SpecAugment, which operates directly on spectrograms.

In order to address the class imbalance, class weight balancing was applied using the compute_class_weight utility from Scikit-learn. This technique adjusts the loss function by assigning higher penalties to minority classes, thus encouraging the model to better handle imbalanced datasets. Early experiments showed that this technique did enhance performance and was applied to all categorical models both during tuning and testing phases. The specific class weights computed for each architecture, seed and fold are provided in Appendix A.

### Ethics declaration

The project adheres to the ethical and professional considerations outlined by the British Computer Society’s (BCS) Code of Conduct and confirms to the ARRIVE guidelines for animal research.

Prior to the data collection phase in this project, ethical approval was sought from the University of Essex for which the preliminary phase of this research was performed. The proposed methods for collection of feline vocalisations were approved with little concerns. The same was reported by^11^ when seeking approval from the Swedish Ethical Review Authority, who likewise confirmed that observations of privately owned cats in their home environment do not require further ethical approval.

Nevertheless, ethical compliance was safeguarded by providing all participants with information outlining their rights to withdraw, data anonymisation, consent for research use, and their data subject rights (Appendix B). This ensures the privacy of the individuals is protected, in compliance with the General Data Protection Regulation (GDPR). This is equally relevant to any human voices accidentally captured during PAM in future work, which are to be excluded from processing^47,62^.

**Table 10:** Categorical results (kitten, adult, senior) on downstream learning task from feature embeddings extracted with VGGish, Perch, and YAMNet. Abbreviations: Acc. (W) = Weighted Accuracy; Acc. = Accuracy; D. (s) = Duration in seconds. Accuracy, Precision, Recall, and F1-Score values have been calculated with macro-averaging to account for data imbalance.

ID	Network	Loss	Acc. (W)	Acc.	Precision	Recall	F1-Score	Kitten Acc.	Adult Acc.	Senior Acc.	D. (s)
A	VGGish	0.76	0.72	0.74	0.72	0.73	**0.72**	0.82	0.71	0.70	54
B	Perch	2.46	0.63	0.60	0.63	0.63	0.60	0.53	0.68	0.60	162
C	YAMNet	5.58	0.56	0.55	0.54	0.56	0.54	0.53	0.59	0.53	158

**Table 11:** Binary results (kitten, senior) on downstream learning task from feature embeddings extracted with VGGish, Perch, and YAMNet. Abbreviations: Acc. (W) = Weighted Accuracy; Acc. = Accuracy; D. (s) = Duration in seconds. Accuracy, Precision, Recall, and F1-Score values have been calculated with macro-averaging to account for data imbalance.

ID	Network	Loss	Acc. (W)	Acc.	Precision	Recall	F1-Score	Kitten Acc.	Senior Acc.	D. (s)
D	VGGish	0.16	0.95	0.95	0.91	0.95	**0.93**	0.95	0.95	52
E	Perch	0.55	0.91	0.88	0.89	0.91	0.87	0.79	0.96	70
F	YAMNet	1.49	0.88	0.84	0.84	0.86	0.84	0.74	0.93	68

## Results and discussion

This section presents the quantitative results of this study, which aims to answer whether we can use deep learning to accurately identify a feline’s age based on their vocalisation. All results consider macro-averaged metrics for a more balanced view of the model’s effectiveness, with an additional weighted accuracy presented for completeness. The macro-averaged accuracy focuses equally on all classes, while the weighted accuracy accounts for class imbalances by giving more weight to frequently occurring classes. To guide the discussion, the models presented in the tables in this section have been given an alphabetical identifier (**ID**).

### Performance of transfer learning models

In this study, we evaluated the performance of VGGish, YAMNet, and Perch models as feature extractors on a downstream MLP neural network learning task. The best-performing model for categorical classification was obtained with VGGish embeddings (***Model A***), achieving an F1-score of **72%** and accuracy of **74%**. For binary classification (kitten vs. senior), the downstream task with VGGish embeddings (***Model D***) achieved an F1-score of **93%** and accuracy of **95%**. All models’ performance is presented in Tables 10 for categorical results and 11 for binary results. The column Duration (s) refers to the total runtime in seconds to build the 5 seed * 4 fold models and to aggregate the results. It has to be noted that code was not executed in isolated environments, potentially affecting the duration. Nevertheless, the recorded runtime provides a guideline for computational costs while we can further refer to the benchmark by^31^ presented in Table 3.

**Fig. 3:**
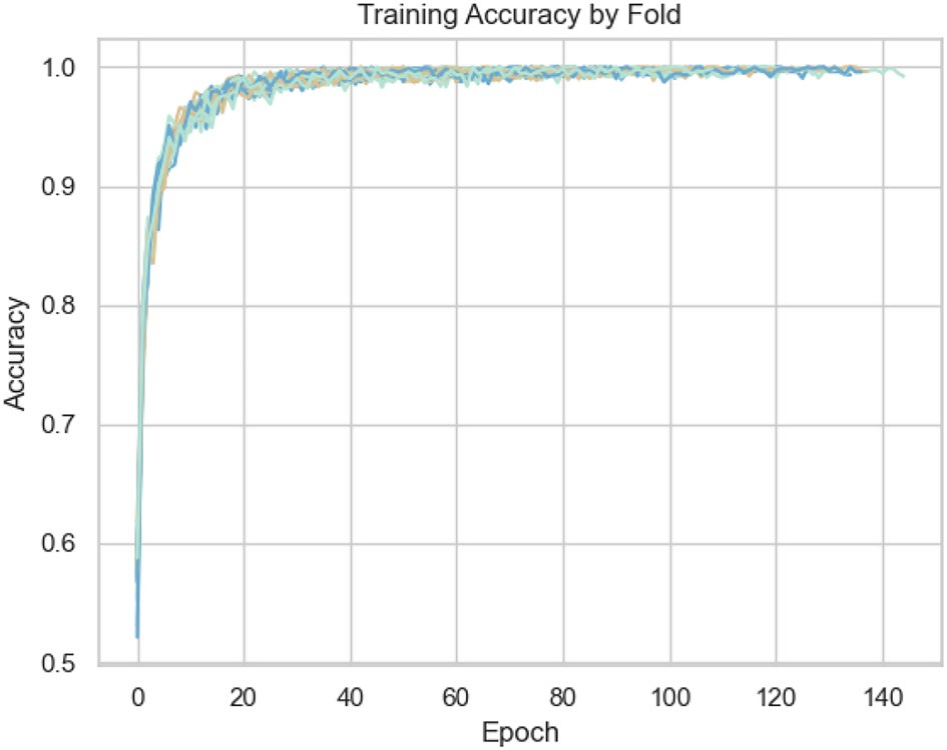
YAMNet Training accuracy over 5 seeds * 4 folds (20 runs total).

**Fig. 4:**
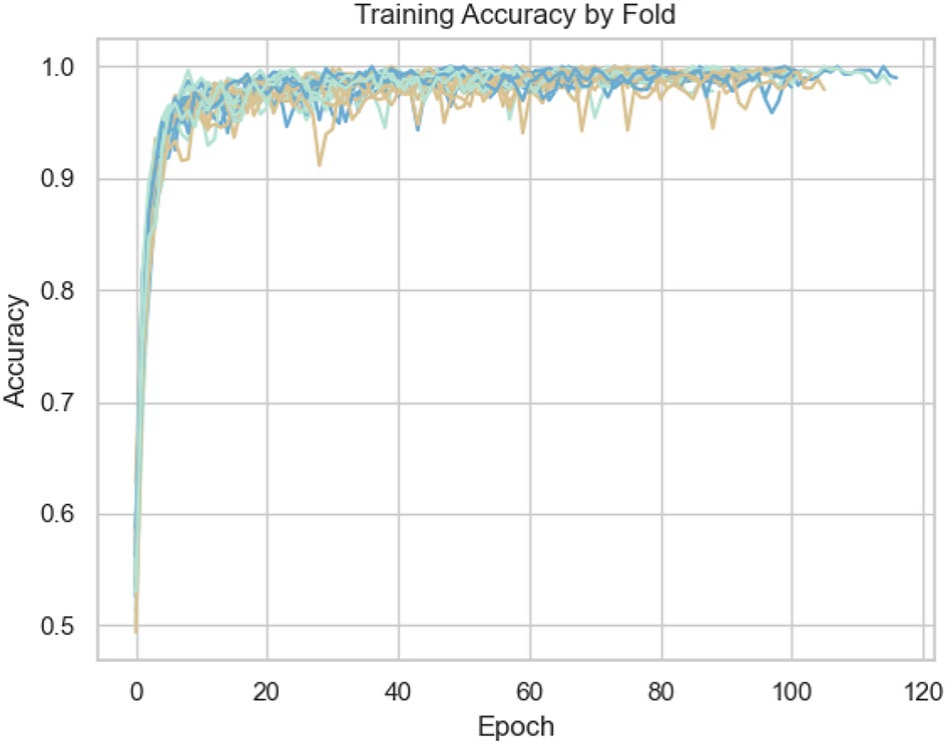
Perch Training accuracy over 5 seeds * 4 folds (20 total).

**Fig. 5:**
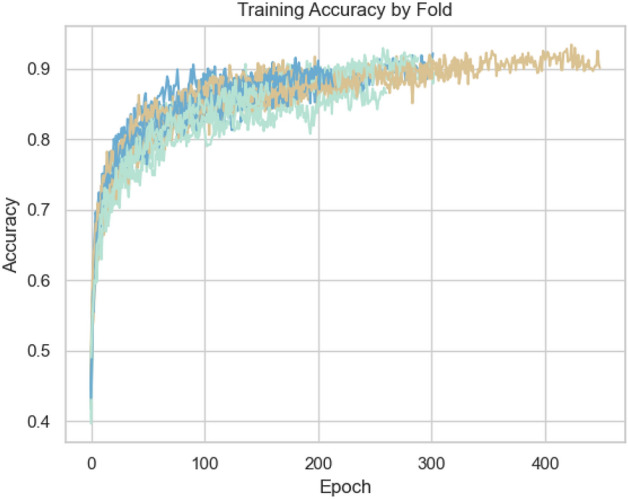
VGGish Training accuracy over 5 seeds * 4 folds (20 total).

Perch and YAMNet embeddings performed significantly worse than VGGish, particularly on the kitten class, and required substantially more computational resources (reflected in tables 3, 10, and 11). The performance issues observed with Perch and YAMNet were compounded by rapid training convergence followed by overfitting (Figs. 3 and 4). Despite attempts to regularise with dropout layers, overfitting remained a challenge. VGGish exhibited a more gradual learning curve (Fig. 5), though some overfitting was still observed, and further improvements in regularisation and data augmentation are needed.

### Comparison with previous work

Our results show that VGGish significantly outperformed both Perch and YAMNet in detecting age-related vocalisation changes, despite prior studies demonstrating Perch’s superiority in broader animal classification tasks^31,37^. Perch’s 5-second embedding window likely mismatched with our shorter 0.72-second vocal samples, limiting its ability to capture subtle age-related features. Similarly, YAMNet, optimised for general sound event detection, struggled to extract the finer details required for this task. By contrast, VGGish’s embedding window - which is shorter than Perch’s - and pre-training on more diverse sound datasets than YAMNet, made it better suited for identifying the nuanced changes in vocalisations associated with age.

To compare our approach against previous studies specifically aimed at age estimation, we may refer back to Table 1. Compared to the works highlighted in Table 1, our study presents several unique contributions and insights:

- **Open-Source Dataset and Configured Pipeline**: Unlike^13^ and^7^, which do not mention dataset availability, our study introduces the first publicly available feline vocalisation dataset tailored for age estimation. This promotes reproducibility and encourages further research in this field.
- **Novelty in Feline Research**: This study represents the first effort to develop a deep learning pipeline specifically for age prediction in domestic cats, filling a gap in bioacoustics and animal behaviour research.
- **Advanced Methodologies**: While previous works such as^13^ and^14^ relied on simpler models (e.g., SVMs and statistical regression models), our study applies state-of-the-art deep learning techniques and transfer learning models. Our methodologies can be repurposed, improved, and integrated into practical applications, such as a mobile app for veterinary or conservation use with adaptability to other species.
- **Competitive Accuracy**: We obtained competitive performance, establishing a strong baseline for future studies.
- **Human Comparison**: While human age estimation studies like^8^ and^9^ achieved high accuracy, they benefited from large-scale datasets (e.g., Mozilla Common Voice). Non-human datasets, including ours, are significantly smaller, yet our results demonstrate the potential for robust age prediction in resource-limited scenarios.
- **Comprehensive Transfer Learning Evaluation**: Unlike prior comparative analyses (e.g.,^31,37^) that found Perch to outperform other models in broad classification tasks, our evaluation reveals that VGGish is better suited for detecting subtle, age-related changes in feline vocalisations. VGGish outperforming YAMNet is in agreement with^32,33,36^, and although with minimal difference it disagrees with^35^.

These findings underscore the potential of deep learning pipelines to advance vocal age prediction across species, particularly in non-human animals where data limitations often pose significant challenges, while also highlighting the importance of selecting models suited to the specific requirements of a given task.

**Fig. 6:**
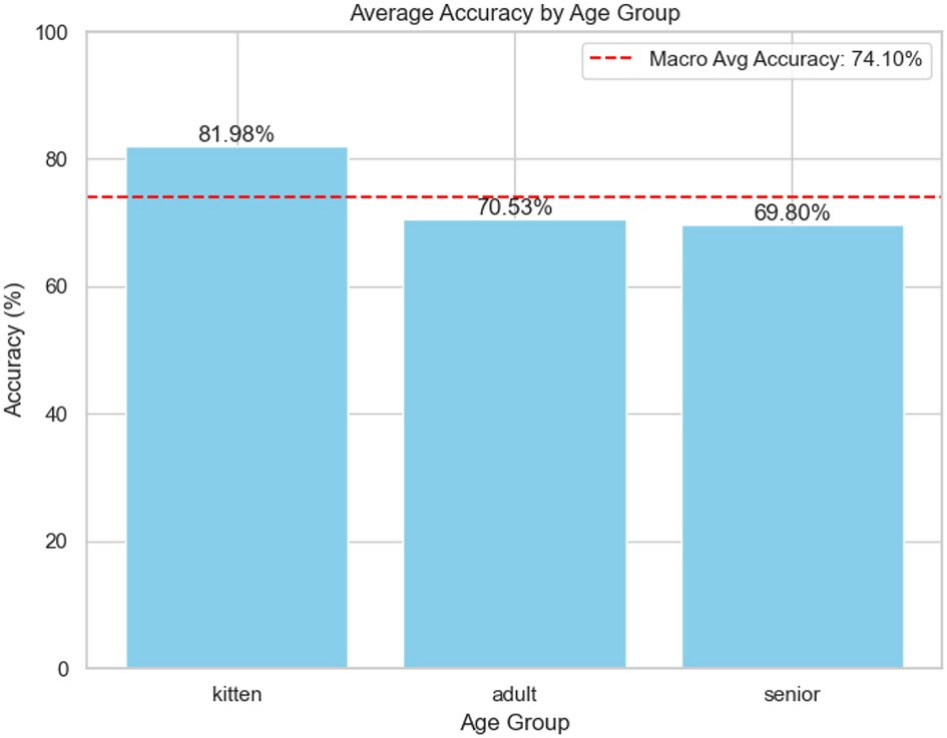
Categorical results per age group for Model A.

**Fig. 7:**
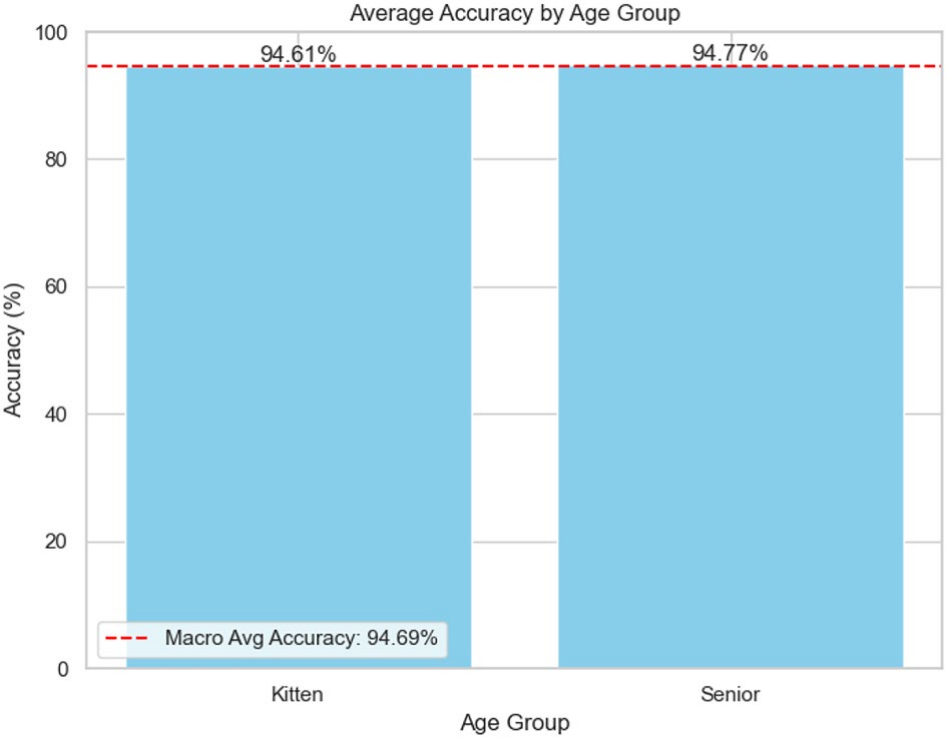
Binary results per age group for Model D.

**Fig. 8:**
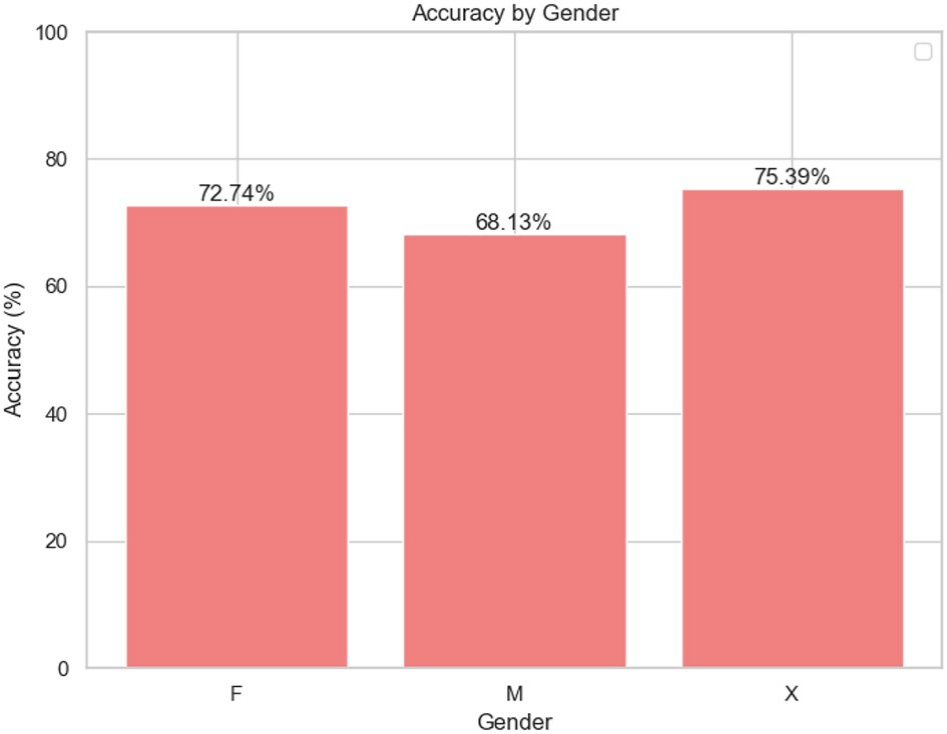
Accuracy by gender for Model A. X = Unknown Gender.

### Visual analysis

**Fig. 9:**
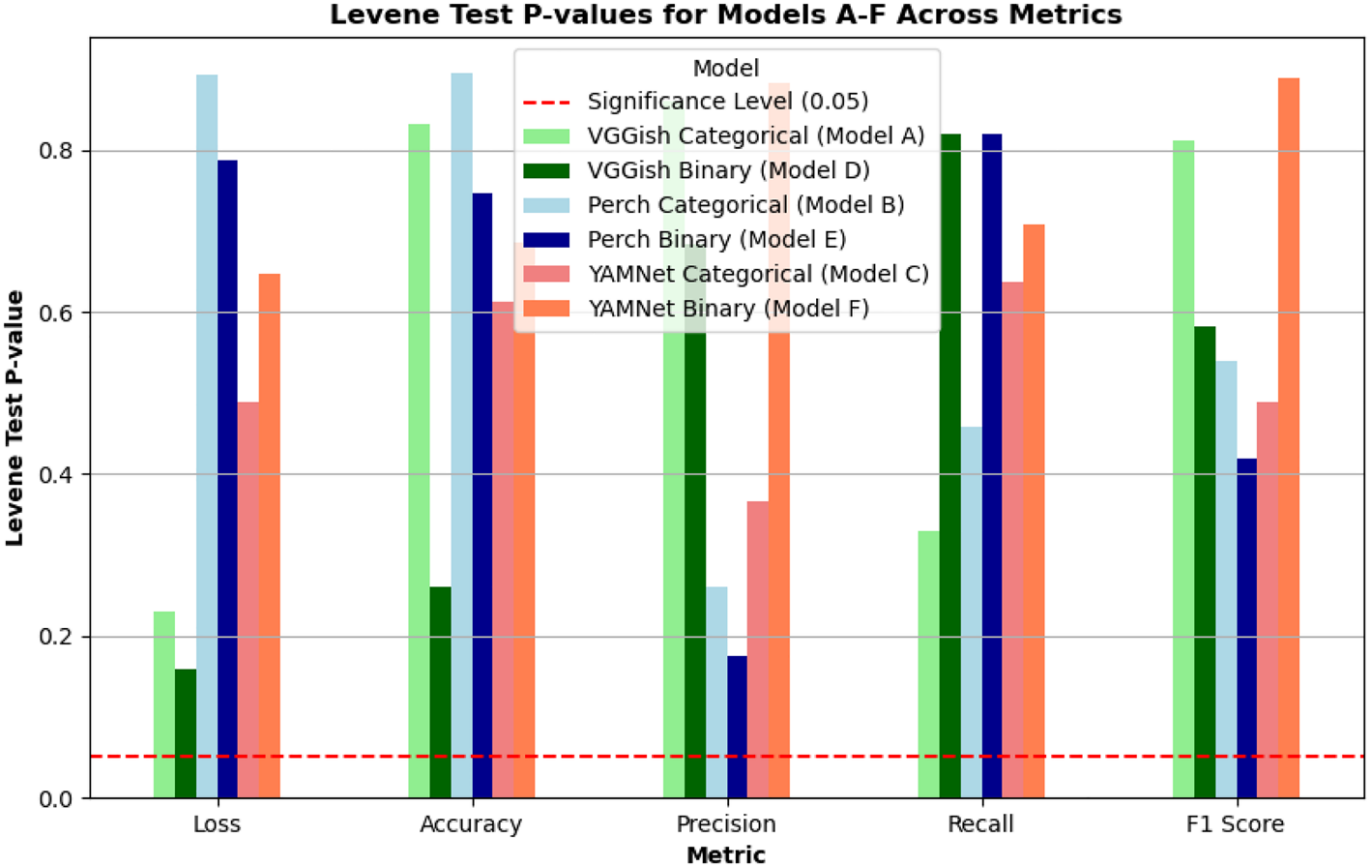
Levene’s test values for statistical significant variance between runs. With a significance level of 0.05, according to these tests none of our models present statistically significant variance in our results estimate.

**Fig. 10:**
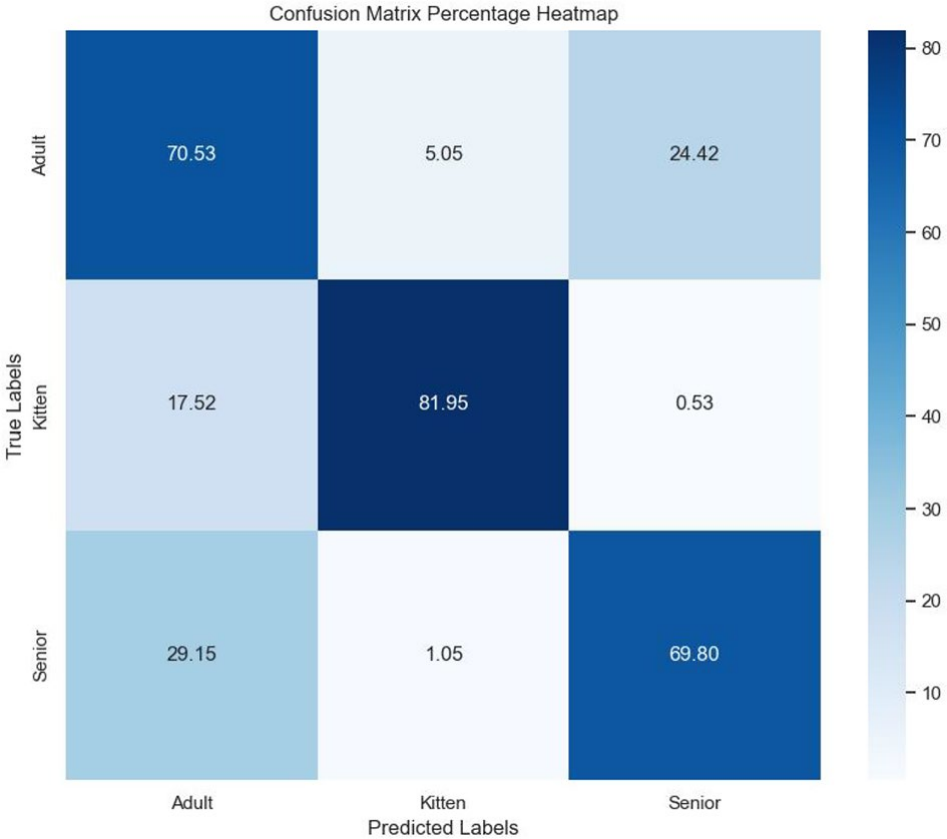
Confusion matrix Model A.

Figures 6 and 7 show the class-based results for the best categorical and binary models (VGGish Models A and D), respectively. Additionally, Fig. 8 presents accuracy by gender, though no significant trends were observed. Levene’s test indicated that performance variance across models was insignificant, suggesting robust estimates to real-world applications (Fig. 9). The confusion matrix for Model A (Fig. 10) shows that kittens were rarely misclassified as seniors and vice versa, although there was some confusion between adults and seniors. This is in line with our highly accurate results in the binary experiment. Improving feature extraction through feature importance analysis and engineering, as well as collecting more senior samples, may help reduce categorical misclassification in future work.

**Fig. 11:**
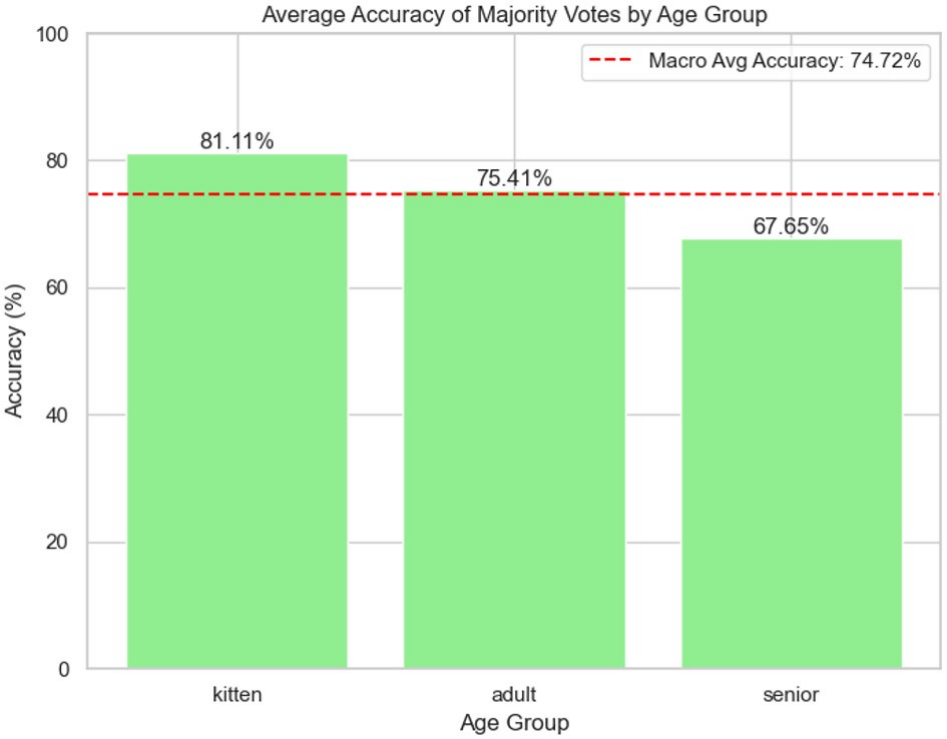
Majority voting values for Model A.

**Table 12:** Mean duration of .wav Files by category.

Category	Mean Duration (seconds)
Kitten	0.78
Adult	0.71
Senior	0.73

**Table 13:** Average number of embeddings per kitten, adult, and senior.

Category	Value
Kitten	10.69
Adult	7.42
Senior	9.00

## Conclusion and limitations

This study successfully validated the hypothesis that feline age can be predicted from vocalisations using deep learning techniques. By leveraging VGGish as the best-performing feature extractor, we demonstrate the feasibility of automated age prediction in domestic felines. Additionally, the novel dataset we collected represents a significant contribution to the field of bioacoustics, providing a foundation for future research in animal age estimation. However, there are some limitations to our approach. The use of overlapping windows for each meow sometimes led to multiple predictions for longer sounds, occasionally producing conflicting results. To address this, we implemented a majority voting mechanism (Fig. 11), which improved accuracy for adult cats but caused a slight decrease for kittens and seniors. This may be due to the differences in meow duration (Table 12) and quantity (Table 13) across age groups, as well as variability in recordings for simultaneously recorded kittens. Future work could explore alternative embedding extraction approaches, such as non-overlapping windows or averaging embeddings, to address these issues. While gender analysis would be valuable for feline age estimation, our dataset with limited gender information prevented detailed gender-specific modeling - though preliminary testing showed minimal impact on estimation accuracy (Fig. 8).

Despite these limitations, our results are promising. Future research could focus on expanding the dataset, particularly for kittens and seniors, and incorporating additional features like a cat’s weight or size, and spectral characteristics of the cat’s meow. Techniques such as few-shot learning, advanced augmentation (e.g., SpecAugment), and deeper hyperparameter tuning could enhance performance. Adding recurrent layers like LSTMs may improve handling of time-series data. Furthermore, while this study focused on evaluating individual transfer learning methods to establish baseline performance, ensemble approaches combining VGGish, YAMNet, and Perch were not explored. Ensemble techniques could enhance robustness of predictions by leveraging the complementary strengths of the models thus we encourage researchers to build on our baseline.

The potential applications of this work are vast. With further performance improvements and increased age groups, this work could lead to the development of a mobile app for veterinarians, pet shelters, and conservationists to aid in feline age estimation, with the potential to expand to other species. A simple Flask web server deployment along with a production demo of the work can be viewed here for binary classification and here for categorical classification. Whilst demo-applications are provided, we emphasize the need for further rigorous development and extended data collection before applying such a tool in a real-world context such as veterinary age estimation or adoption processes. The harm in misclassifying a feline’s age could affect healthcare plans or owner distress in incorrect life expectancy, we therefore advocate for a multi-model approach to optimise age estimation.

Ultimately, this research aims to support advancements in interspecies communication, where age plays a key role in understanding animal behaviour. This work may offer a pipeline for age prediction that extends to species beyond the domestic felines, though attention must be paid to the differences in vocal behaviour across species. We also wonder whether age recognition could aid efforts in the recognition of individual animals in long-term tracking studies for conservation - a direction worth exploring.

## Supplementary Information

Supplementary Information.

## Data Availability

A GitHub repository including the dataset, all code, and analysis used in this project is available at https://github.com/aster-droide/feline-age-prediction. A clone of the VGGish codebase including the modified inference file for feature extraction used in this project is available from this location. The Flask web server deployment along with a simple production demo of the work can be viewed here for binary production and here for categorical.

## Data availability

A GitHub repository including the dataset, all code, and analysis used in this project is available at https://github.com/aster-droide/feline-age-prediction. A clone of the VGGish codebase including the modified inference file for feature extraction used in this project is available from https://github.com/aster-droide/audioset-thesis-work/blob/main/audioset/vggish/vggish_inference_demo.py. The Flask web server deployment along with a simple production demo of the work can be viewed at https://github.com/aster-droide/age-prediction-demo-binary for binary production and https://github.com/aster-droide/age-prediction-demo-categorical for categorical.
