# Supplementary material for: A deep learning pipeline for age prediction from vocalisations of the domestic feline
Source: Sci Rep. 2025 Oct 3;15:34565. doi: 10.1038/s41598-025-17986-z (PMC12494702; doi:10.1038/s41598-025-17986-z)
Supplement: Supplementary file 1 — Supplementary Information. [file 41598_2025_17986_MOESM1_ESM.pdf]

Appendix A Class Weights per Architecture, Seed, and Fold for Downstream MLP Task

Table A1: YAMNet Class Weights for Categorical Classification per Seed and Fold for Downstream MLP Task

Seed	Fold 1			Fold 2			Fold 3			Fold 4		
	Kitten	Adult	Senior	Kitten	Adult	Senior	Kitten	Adult	Senior	Kitten	Adult	Senior
7270	1.76	0.69	1.01	1.81	0.64	1.12	1.48	0.78	0.96	1.74	0.66	1.10
860	1.73	0.72	0.98	1.79	0.69	1.01	1.56	0.67	1.16	1.66	0.69	1.05
5390	1.73	0.69	1.04	1.55	0.68	1.13	2.01	0.73	0.88	1.57	0.66	1.16
5191	1.73	0.64	1.16	1.49	0.83	0.89	1.54	0.67	1.15	2.09	0.65	1.02
5734	1.93	0.70	0.95	1.60	0.70	1.06	1.55	0.78	0.93	1.71	0.61	1.29

Weights were calculated dynamically with sklearn's compute_class_weight() for each seed and fold based on class distribution for the downstream categorical learning task on the YAMNet architecture.

Table A2: VGGish Class Weights for Categorical Classification per Seed and Fold for Downstream MLP Task

Seed	Fold 1			Fold 2			Fold 3			Fold 4		
	Kitten	Adult	Senior	Kitten	Adult	Senior	Kitten	Adult	Senior	Kitten	Adult	Senior
7270	1.72	0.64	1.16	1.71	0.77	0.90	1.68	0.65	1.16	1.65	0.69	1.06
860	1.97	0.66	1.02	1.64	0.71	1.02	1.66	0.66	1.12	1.52	0.71	1.08
5390	1.59	0.68	1.11	1.90	0.71	0.94	1.83	0.62	1.20	1.46	0.76	1.00
5191	1.77	0.64	1.16	1.79	0.66	1.08	1.59	0.68	1.11	1.68	0.77	0.9
5734	1.81	0.66	1.08	1.55	0.71	1.05	1.56	0.66	1.18	1.98	0.71	0.91

Weights were calculated dynamically with sklearn's compute_class_weight() for each seed and fold based on class distribution for the downstream categorical learning task on the VGGish architecture.

Table A3: Perch Class Weights for Categorical Classification per Seed and Fold for Downstream MLP Task

Seed	Fold 1			Fold 2			Fold 3			Fold 4		
	Kitten	Adult	Senior	Kitten	Adult	Senior	Kitten	Adult	Senior	Kitten	Adult	Senior
7270	1.85	0.73	0.92	1.81	0.62	1.22	1.77	0.66	1.08	2.01	0.62	1.13
860	2.25	0.68	0.92	1.48	0.68	1.17	1.90	0.61	1.22	1.87	0.66	1.05
5390	1.75	0.72	0.95	2.13	0.60	1.15	1.59	0.59	1.47	1.91	0.72	0.93
5191	1.60	0.73	1.00	2.40	0.61	1.06	1.74	0.64	1.17	1.73	0.67	1.07
5734	1.68	0.72	0.98	2.17	0.59	1.17	1.51	0.73	1.03	2.09	0.60	1.16

Weights were calculated dynamically with sklearn's compute_class_weight() for each seed and fold based on class distribution for the downstream categorical learning task on the Perch architecture.

Table A4: Perch Class Weights for Binary Classification per Seed and Fold for Downstream MLP Task

Seed	Fold 1		Fold 2		Fold 3		Fold 4	
	Kitten	Senior	Kitten	Senior	Kitten	Senior	Kitten	Senior
7270	1.54	0.74	1.15	0.88	1.39	0.78	1.41	0.77
860	1.22	0.85	1.68	0.71	1.17	0.88	1.42	0.77
5390	1.22	0.85	1.45	0.76	1.63	0.72	1.20	0.86
5191	1.22	0.85	1.48	0.75	1.28	0.82	1.45	0.76
5734	1.62	0.72	1.33	0.80	1.23	0.84	1.32	0.80

For binary classification, Perch was the only architecture that benefited from class weights. Weights were calculated dynamically with sklearn's compute_class_weight() for each seed and fold based on class distribution for the downstream binary learning task on the Perch architecture.

Appendix B Consent & Confirmation Email including Data Subject Rights for Data Collection

Dear Cat Owner,

Thank you very much for your valuable contribution to our feline age prediction project. We have successfully received the data you have provided.

As part of our commitment to privacy and data management, we have assigned a unique identifier number to your contribution: [XXX]. Please keep this number safe, as it will be required if you wish to withdraw your data from the study. Any further contributions will automatically be tied to this identifier.

By sending us this data, you are consenting to its use for research purposes as part of a deep learning tool for age estimation of the domestic cat. Our study aims to develop a non-invasive method to determine a cat's age through vocalisations. This groundbreaking approach promises significant benefits in veterinary care, can aid rescue centres in creating accurate adoption profiles, and has potential implications for understanding the age demographics of feral cats.

Please be assured that all data will be handled confidentiality. The sound clips you have provided will be anonymised, ensuring that no personal identifiers are linked to the data in any of our analyses or reports.

We also want to inform you of your rights in this process:

Right to Withdraw: You have the right to withdraw your consent and participation at any point without any adverse consequences. Should you decide to withdraw, please inform us via email and provide your unique identifier number. With this number, we will be able to locate and promptly remove your data from our study.

Data Subject Rights: As a contributor, you are entitled to request information about how your data is being used, and to see the data you have provided. If you wish to see the data you have provided, please contact us with your unique identifier number. We will use this number to retrieve and share your specific data with you.

Furthermore, to protect your privacy, your email address and any other personal contact information will be deleted from our records once the data collection phase of the project is completed.

If you have any questions or require further information, please do not hesitate to contact us.

Thank you once again for your contribution to this research. Your participation is invaluable in helping us gain insights into the development of age-related vocalisation patterns in domestic cats.

Best regards,

Astrid van Toor

MSc Artificial Intelligence
University of Essex
